# Transparency of COVID-19-Related Research in Dental Journals

**DOI:** 10.3389/froh.2022.871033

**Published:** 2022-04-06

**Authors:** Ahmad Sofi-Mahmudi, Eero Raittio

**Affiliations:** ^1^Seqiz Health Network, Kurdistan University of Medical Sciences, Sanandaj, Iran; ^2^Cochrane Iran Associate Centre, National Institute for Medical Research Development, Tehran, Iran; ^3^Institute of Dentistry, University of Eastern Finland, Kuopio, Finland

**Keywords:** COVID-19, dentistry, dental research, data, FAIR data principles, open science, reproducibility, transparency

## Abstract

**Objective:**

We aimed to assess the adherence to transparency practices (data availability, code availability, statements of protocol registration and conflicts of interest and funding disclosures) and FAIRness (Findable, Accessible, Interoperable, and Reusable) of shared data from open access COVID-19-related articles published in dental journals available from the Europe PubMed Central (PMC) database.

**Methods:**

We searched and exported all COVID-19-related open-access articles from PubMed-indexed dental journals available in the Europe PMC database in 2020 and 2021. We detected transparency indicators with a validated and automated tool developed to extract the indicators from the downloaded articles. Basic journal- and article-related information was retrieved from the PMC database. Then, from those which had shared data, we assessed their accordance with FAIR data principles using the F-UJI online tool (f-uji.net).

**Results:**

Of 650 available articles published in 59 dental journals, 74% provided conflicts of interest disclosure and 40% funding disclosure and 4% were preregistered. One study shared raw data (0.15%) and no study shared code. Transparent practices were more common in articles published in journals with higher impact factors, and in 2020 than in 2021. Adherence to the FAIR principles in the only paper that shared data was moderate.

**Conclusion:**

While the majority of the papers had a COI disclosure, the prevalence of the other transparency practices was far from the acceptable level. A much stronger commitment to open science practices, particularly to preregistration, data and code sharing, is needed from all stakeholders.

## Introduction

The foundation of science is to generate knowledge from reproducible findings [1]. Because of the failure to reproduce previous research, the open science movement emerged in recent years [2]. The movement's principal focus is on making science more accessible and trustworthy. Data and code sharing, protocol registration and funding and conflicts of interest (COI) disclosures are considered important features of open science and crucial when assessing the credibility of certain scientific findings [2, 3]. Science without credibility and quality would do a major human, societal, and economic disservice [4].

COVID-19 pandemic launched an incredibly fast and voluminous avalanche of scientific publications across the different fields of science. Related to the COVID-19 publications there has been a considerable amount of discussion and examples related to poor or suboptimal data, methods and other scientific practices [5–8]. Unsurprisingly, there have been signs of fabricated data, poor data and methods and conflicts of interests in the scientific literature related to the COVID-19 pandemic, which has resulted in a great number of retracted papers (208 as of the end of January 2022) [9]. Probably, if transparent scientific practices, like data and code sharing, would be more like the rule than the exception in biomedical research, pre- and post-publication evaluation of the credibility of studies could be much easier [10, 11].

The COVID-19 pandemic and its implications for dental services, oral health, oral health-related behaviors and dental education have received great attention from the dental research community. Nevertheless, to date, only one paper related to dentistry or published in dental has been retracted from the Journal of Oral and Maxillofacial Pathology [9]. However, meta-research about reporting, methods or reproducibility in COVID-19-related dental research has been limited. Particularly sharing data with adequate quality would allow reanalyses of findings [10].

Our aim was to assess the uptake of transparent scientific practices (data sharing, code sharing, COI disclosures, funding disclosures, and protocol registration) from open access full-text COVID-19-related articles published in dental journals available from the Europe PubMed Central (EPMC) database. We also assessed the FAIRness (Findable, Accessible, Interoperable, and Reusable) of data from those articles that have shared their data. We mapped transparency practices across publication years, publishers, and journals.

## Materials and Methods

The protocol of this study was published beforehand on the Open Science Framework (OSF) website (osf.io/f53eq). All code and data related to the study were shared *via* its OSF repository (osf.io/yx4ce) at the time of submission of the manuscript.

### Data Sources and Study Selection

We searched for open access COVID-19-related articles from dental journals available in the EPMC database. We reviewed dental journals based on a list of PubMed-indexed dental journals provided by the National Library of Medicine catalog [12]. We restricted our search to papers in English and considered papers published from 01-01-2020 to 31-12-2021. We restricted our search to articles that had a variant of the COVID-19 keyword in their title, keywords, or results to grab only the most likely relevant papers as, during the COVID-19 pandemic, many unrelated papers to COVID-19 have been seen to use COVID-19 in their introduction, methods, discussion, or abstract. This enabled us to bypass screening for COVID-19-related papers and decrease the human labor for our research. We confirmed our approach by choosing a random sample of 100 papers and of those, all were relevant to COVID-19.

The search query in the EPMC database was as follows:

- “((ISSNs for all dental journals) AND (COVID-19 query for title, keywords, and results) AND (SRC:“MED”) AND (LANG:“eng” OR LANG:“en” OR LANG:“us”) AND (FIRST_PDATE:[2020-01-01 TO 2021-12-31]) AND ((IN_EPMC:y) OR (OPEN_ACCESS:y)) AND (PUB_TYPE:“Journal Article” OR PUB_TYPE:“research-article” OR PUB_TYPE:“rapid-communication” OR PUB_TYPE:“product-review”)”

The COVID-19 query is available in Appendix 1.

We downloaded all identified available records in XML full-text format (for full-text evaluation) using the metareadr package from the database [13].

### Data Extraction and Synthesis

We assessed adherence to five transparent practices:

- data availability,
- code availability,
- COI disclosures,
- funding disclosures,
- and statements of protocol registration.

To do so, we used a validated and automated text-mining tool developed by Serghiou et al. [3] suitable to identify these five transparent practices from articles in XML format from the EPMC database.

Basic journal- and article-related information (publisher, publication year, citations to article, and journal name) were retrieved from the EPMC. We downloaded additional information about journal impact factors from the Journal Citation Reports 2021.

For assessing the FAIRness of shared data [14], we first manually confirmed that articles had actually shared the data and then we used the F-UJI tool [15]. The output of this tool is four individual scores for each component of the FAIR principle, as follows:

- Findability: out of 7;
- Accessibility: out of 3;
- Interoperability: out of 4; and,
- Reusability: out of 10.

In addition, as a summary score, it provides the percentage out of a maximum score of 24 (sum of the four components).

### Data Analysis

We used R v4.1.1. [16] for searches, data handling, analysis and reporting. The searches and data export from the Europe PMC were conducted with the europepmc package [17]. Transparency indicators from the available full-texts were extracted with the rtransparent package [3]. Comparison between 2020 and 2021 in transparency indicators by journal- and article-related information were reported using descriptive tabulations and graphical illustrations, for instance using the ggplot2 package [18]. We used the numbers for sensitivity and specificity of the rtransparent package [3] to generate 95% confidence intervals (CIs) for our prevalence estimates of the transparency indicators with the epiR package [19]. We also provided monthly trends for transparency indicators, to check whether there were any trends in transparency practices over time. We used Chi-square for comparing indicators between years and the Wilcoxon rank sum test to test whether there was a relationship between transparency indicators and JIFs.

## Results

The total number of COVID-19-related papers (open access and non-open access) was 1,038, of which full texts of 657 papers (63.3%) were accessible *via* the EPMC. Of those, 337 were published in 2020 and 320 in 2021. However, as we could not retrieve seven full texts from the EPMC database due to technical issues, our final sample included 650 full-text articles (Supplementary Figure 1).

These articles were published in 59 journals of which the top five were British Dental Journal (*n* = 69), Journal of Dental Education (*n* = 59), British Journal of Oral and Maxillofacial Surgery (*n* = 51), Oral Diseases (*n* = 47), and BMC Oral Health (*n* = 40). The mean and the median of the number of citations to these articles were 7.7 [standard deviation (SD) = 43.15] and 2 [interquartile range (IQR) = 5]. The highest cited article with 854 citations was an article published in the International Journal of Oral Sciences in 2020 [20].

About three–fourths of the articles (*n* = 478) had a statement to disclose COI (73.5%, 95% CI: 70.0–76.8%). Articles published in 2021 had a higher rate of COI disclosure (79.6 vs. 67.8%, *P* < 0.001). About four–tenths of the references had a funding statement (*n* = 261) (40.2%, 95% CI: 36.5–43.0%). Articles in 2021 had a funding statement more frequently than 2020 articles (49.1 vs 31.6%, *P* < 0.001). Less than 1 in 20 of the articles (*n* = 27) were registered beforehand (4.2%, 95% CI: 2.9–6.0%) and the rate was six times higher in 2021 compared with 2020 (7.2 vs. 1.1%, *P* < 0.001). Regarding data and code availability, as we chose the software to have low specificity to be as sensitive as possible, we got some false positives. At first, we got four papers with shared data and none with shared code. After checking manually, we omitted four false positives from the shared data papers. One of them used the journal's template: “The data that support the findings of this study are openly available in [repository name] at [DOI]” [21]. Hence finally, one paper had shared data (0.2%). This article was published in BMC Oral Health in 2021 [22].

A total of 117 articles were published in journals with no impact factor. Papers that had a COI disclosure, funding statement and were registered tend to be published in journals with higher JIF (*P* = 0.003, *P* < 0.001, and *P* = 0.007, respectively). Detailed information is illustrated in Table 1.

**Table 1:** Mean and median of Journal Impact Factor (JIF) for papers with different levels of transparency.

**Measurement**	**Mean (SD)**		**Median (IQR)**		***P*-value**
	**With**	**Without**	**With**	**Without**	
COI disclosure	2.898 (1.2981)	2.636 (1.0726)	2.650 (1.678)	2.264 (1.162)	0.003
Funding statement	3.234 (1.4707)	2.538 (0.9586)	2.757 (1.309)	2.264 (1.860)	<0.001
Registration	3.246 (1.0723)	2.816 (1.2572)	3.542 (0.8925)	2.512 (1.6160)	0.007

*P-value based on the Wilcoxon rank sum test*.

*COI, Conflict of interest; SD, Standard deviation; IQR, Inter-quartile range*.

**Figure 1:**
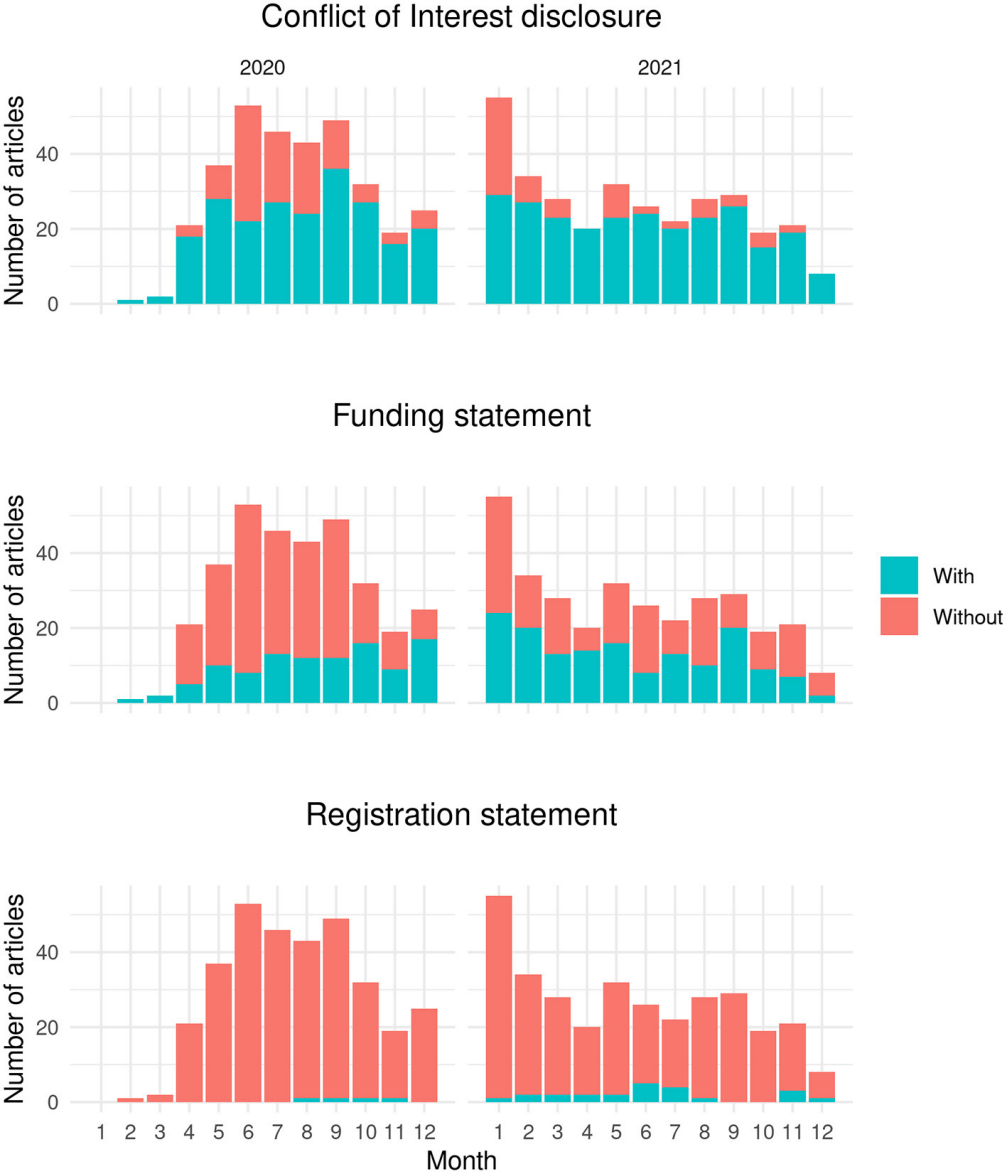
The monthly trend for three transparency indicators, namely conflict of interest (COI) disclosure, funding statement, and registration for 2020 and 2021.

Figure 1 shows the monthly trend for three transparency indicators. There was an inconsistent trend for the indicators. However, the COI disclosure percentage seemed to be more consistent and showed an increasing trend over time.

FAIRness analysis of the research paper that shared its data showed that it was moderately FAIR (70%) with an overall score of 17 out of 24. The score in each component of FAIRness was as follows: (1) findability: 7/7 (advanced level); (2) accessibility: 2/3 (moderate level); (3) interoperability: 2/4 (initial level); and (4) reusability: 6/10 (advanced level).

## Discussion

Our study showed that the majority of COVID-19-related dental articles had COI disclosure. Funding statements were also quite often included, whereas protocol registration, data and code sharing were rare. Positive trends occurred over time and articles published in journals with higher impact factors showed more favorable transparency practices.

COIs have been a concern in biomedical research for decades, for instance, related to tobacco industry manipulation of research [23]. It has been shown that funded trials may be more positive due to biased interpretation of trial results [24]. In the case of dental research, the effect of industry on dental research has been of great concern [25]. With the special situation of the COVID-19 pandemic and the great uncertainty regarding its treatment and prevention, the importance of declaring COI has increased. COI may shape health policies and systems, e.g., related to oral health care delivery, but also shape the reaction of the health care systems to the pandemic situation [26, 27]. While having a conflict of interest may be inevitable, they may also be hard to recognize [28]. However, even if one has no COIs, it should be declared. Our results showed that whereas the majority of the papers had a COI statement, about one in four still did not have one, and this was on quite a similar level compared to the previous studies on dental literature in 2014 [29] and 2020 [30].

Funding disclosures were less frequent than COI disclosures in the studied articles. One of the possible reasons could be the journal's less strict policy for an obligation of funding disclosures than for COI disclosures. However, evidence indicates that many journals require funding disclosure too [31]. Whilst there are reports of higher proportions for funding disclosure for randomized controlled trials [32, 33], most COVID-19-related research articles have been observational studies, editorials, letters, and reviews, and thus it is logical that most research did not receive funding which would have affected the content of the articles. However, we should bear in mind that even if external funding was not received, it should be stated in order to increase transparency in science.

Less than five percent of papers were registered beforehand. Pre-registration does not only relate to randomized controlled trials or reviews but observational, *in-vivo*, and *in-vitro* studies, or in other words, almost all studies, could and should be pre-registered. Currently, there are plenty of platforms for study registration including the OSF which support registering any type of research study. It has also been argued that pre-registration could improve the interpretability and credibility of findings [34]. Manifold publication of similar studies, seen also during the COVID-19 pandemic [5], could be prevented to some extent with preregistration and thus the research waste would be decreased.

Surprisingly, in our sample, only one study shared its data and no study shared code. With sharing data and codes, the replicability of the research could be achieved and also secondary analysis of the data could be possible. In addition, sharing data and code would enable the detection of quite common errors in data and analyses [10, 35]. Sharing COVID-19 research data is a challenge, which several studies have addressed [36–38], however, 1 in 650 what we saw in our sample is not near the optimum. We encourage oral health researchers to share all their data and codes in adherence to FAIR principles [14] in order to increase the transparency, accessibility, and replicability of their research. Journal policies should also emphasize data and code sharing and publishing at least the metadata of the research data.

Already in January 2020, over 100 organizations, including journals, publishers, funders, universities and other institutions, signed a statement that helped to ensure free access to research data, tools, and other information related to COVID-19. Later, also other significant initiatives to support such goals emerged worldwide. According to Vuong et al. [39], these transparency practices facilitated multi-discipline collaboration between stakeholders with similar interests in producing innovative solutions to the pandemic, for instance the incredible fast development of COVID-19 vaccines. However, our findings imply that, apart from the high proportion of open access articles from the total number of COVID-19-related dental articles, the contribution of dental researchers to help fight the pandemic by the means of data or code sharing was trivial. We hope sharing data and other research material would be more often seen as a transparent practice advancing collaborative science rather than a painstaking idealistic task.

### Limitations

The study sample was restricted to open access articles in the EPMC database which may not correspond to all COVID-19-related studies published in dental journals. However, as two–thirds of COVID-19-related papers have been open access, this did not diminish the strength of our interpretations considerably. It is also not possible not to evaluate how trustworthy the detected disclosures were. For instance, in one study, the data availability statement included the journals' template without any changes to it. It is also evident that despite validated text-mining algorithms the actual prevalence of transparency practices may have been under- or overestimated in our study.

## Conclusion

Our study showed that while the majority of the papers had a COI disclosure, however the proportion for other transparency indicators was far from the acceptable level. In the case of data and code sharing, only one study shared its data with a moderate FAIR level and no study shared code. This could be an alarming sign that journal editors should consider transparency practices when deciding on a research paper to be published in their journal. We hope our findings encourage the dental research community to a much stronger commitment to open science practices. It would be a major service for the public and societies [4, 40].

## Data Availability Statement

The datasets presented in this study can be found in online repositories. The names of the repository/repositories and accession number(s) can be found below: Open Science Framework (OSF) storage (osf.io/yx4ce), the figshare repository for the study (https://doi.org/10.6084/m9.figshare.19131245), and GitHub repository for the study (https://github.com/choxos/covid-dental-transparency/).

## Author Contributions

AS-M: conceptualization, methodology, validation, formal analysis, investigation, resources, data curation, writing–original draft, writing–review and editing, visualization, and supervision. ER: conceptualization, methodology, investigation, writing–original draft, and writing–review and editing. All authors contributed to the article and approved the submitted version.

## Conflict of Interest

The authors declare that the research was conducted in the absence of any commercial or financial relationships that could be construed as a potential conflict of interest.

## Publisher's Note

All claims expressed in this article are solely those of the authors and do not necessarily represent those of their affiliated organizations, or those of the publisher, the editors and the reviewers. Any product that may be evaluated in this article, or claim that may be made by its manufacturer, is not guaranteed or endorsed by the publisher.
