# Supplementary material for: Transparency of COVID-19-Related Research in Dental Journals
Source: Front Oral Health. 2022 Apr 6;3:871033. doi: 10.3389/froh.2022.871033 (PMC9019132; doi:10.3389/froh.2022.871033)
Supplement: Supplementary file 1 [file Table_1.DOCX]















*Consider, if feasible to do so, reporting the number of records identified from each database or register searched (rather than the total number across all databases/registers).

**If automation tools were used, indicate how many records were excluded by a human and how many were excluded by automation tools.

*From:*  Page MJ, McKenzie JE, Bossuyt PM, Boutron I, Hoffmann TC, Mulrow CD, et al. The PRISMA 2020 statement: an updated guideline for reporting systematic reviews. BMJ 2021;372:n71. doi: 10.1136/bmj.n71

For more information, visit: <http://www.prisma-statement.org/>
